# Insights into the Regulation of Ciliary Disassembly

**DOI:** 10.3390/cells10112977

**Published:** 2021-11-01

**Authors:** Maulin M. Patel, Leonidas Tsiokas

**Affiliations:** Department of Cell Biology, University of Oklahoma Health Sciences Center, Oklahoma, OK 73104, USA; leonidas-tsiokas@ouhsc.edu

**Keywords:** cilia, cilia disassembly, ciliopathies

## Abstract

The primary cilium, an antenna-like structure that protrudes out from the cell surface, is present in most cell types. It is a microtubule-based organelle that serves as a mega-signaling center and is important for sensing biochemical and mechanical signals to carry out various cellular processes such as proliferation, migration, differentiation, and many others. At any given time, cilia length is determined by a dynamic balance of cilia assembly and disassembly processes. Abnormally short or long cilia can cause a plethora of human diseases commonly referred to as ciliopathies, including, but not limited to, skeletal malformations, obesity, autosomal dominant polycystic kidney disease, retinal degeneration, and bardet-biedl syndrome. While the process of cilia assembly is studied extensively, the process of cilia disassembly and its biological role(s) are less well understood. This review discusses current knowledge on ciliary disassembly and how different cellular processes and molecular signals converge to carry out this process. This information will help us understand how the process of ciliary disassembly is regulated, identify the key steps that need further investigation, and possibly design therapeutic targets for a subset of ciliopathies that are causally linked to defective ciliary disassembly.

## 1. Introduction

Cilia are antenna-like organelles present on almost all cell types. They are microtubule-based structures that protrude from the cell surface [1]. The primary cilium possesses a specialized membrane called the ciliary membrane that sheaths its structure and compartmentalizes its associated signaling complexes [2,3]. In general, cilia are thought to act as sensors of different biochemical and mechanical cues that mediate cellular processes such as proliferation, differentiation, and cell migration [4,5]. They mediate such function(s) by housing a wide variety of ion channels [6,7,8] and receptors essential for multiple signaling pathways. Some of the most common signaling pathways studied in association with cilia are Hedgehog [9,10], WNT [11,12], NOTCH [13], HIPPO [14,15], TGF, and growth factor signaling pathways [15]. Since cilia act as mega-signaling centers regulating multiple signaling pathways important for various cellular functions [15], proper cilia length regulation is crucial for carrying out these various function(s). A unique feature of primary cilia is that they are formed when cells exit the cell cycle and disassemble when cells enter the cell cycle. Therefore, ciliary length is subject to dynamic regulation depending on the stage of the cell cycle. In resting cells at G0, the cilia assembly pathways are predominant, resulting in fully formed cilia. However, in cycling cells, especially in the S/G2/M phases of the cell cycle, cilia disassembly pathways prevail. Several excellent reviews on cilia assembly, disassembly, or both, have been published previoulsy [16,17,18,19,20,21,22,23,24]. However, in recent years, several major advances have been made, shedding new light on the mechanisms of ciliary disassembly and its biological role(s). Here, we have reviewed and integrated pre-existing knowledge with recent developments, which will be helpful in guiding further investigations toward understanding the process of ciliary disassembly.

## 2. The Primary Cilium: Structure and Types

**Figure 1:**
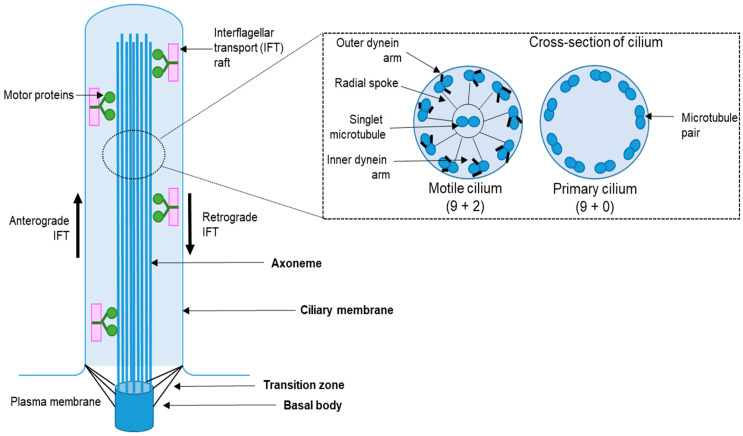
Simplistic overview of cilia structure and types. Structurally, the cilium consists of four main parts,(1) the basal body, (2) the transition zone, (3) the axoneme, and (4) the ciliary membrane. Furthermore, there are two main types of cilium: (1) motile cilium, containing 9 outer microtubules (MTs) doublets and one pair of MT at its center, and (2) nonmotile cilium, which has 9 outer MT doublets but lacks the central MT pair.

Structurally, the cilium comprises four main compartments: (1) the basal body, (2) the transition zone, (3) the axoneme, and (4) the ciliary membrane [25] (Figure 1). The basal body is located at the base of the cilia and is a modified form of the mother centriole from which the axoneme microtubules (MTs) stem out. The axoneme is assembled by a cylindrical arrangement of nine triplet MTs that arises from the mother centriole and gets anchored to the ciliary membrane through transition fibers. The transition zone is a ciliary compartment localized above the basal body and houses the machinery that controls the entry and exit of signaling molecules from the cilia [26,27,28]. The basal body and transition zone further support the structural dynamics of the axonemal MTs during cilia assembly or disassembly. The axoneme is considered as the core of the cilium, and it consists of MTs doublets, of which the positive end is oriented toward the tip of the cilium. Recent evidence also suggests the presence of actin filaments toward the distal end of the axoneme [1]. Along with the elongation of the axoneme, the ciliary membrane also extends and sheaths the growing axoneme and harbors a wide variety of receptors and ion channels that mediate multiple signaling pathways [3,9,10,11,12,13,14,15,29,30].

While the majority of ciliated cells assemble only one cilium (primary cilium), some cells can assemble multiple cilia. Based on their motility and axonemal architecture, cilia are categorized into nonmotile and motile cilia (Figure 1). Primary or nonmotile cilia have axoneme with 9  + 0 MT pattern, where 9 MT doublets are found in cylindrical arrangement with no MT at the center. In contrast, motile cilia have axoneme with a 9  +  2 MT pattern, where 9 MT doublets are found in cylindrical arrangement with 2 MT localized at its center. An exemption to this pattern is the motile cilia in the embryonic node with a 9 + 0 MT configuration [31]. In vertebrates, primary or nonmotile cilia are present on most cell types in a single copy and sense extracellular biochemical and mechanical signals [15]. Motile cilia can be found on some specialized epithelial cells in vertebrates and in unicellular organisms such as *Chlamydomonas* or Tetrahymena. Motile cilia can move or beat in a highly regulated fashion in order to displace or transport fluids (e.g., along the central nervous ependymal lining or surfaces of upper or lower respiratory epithelium) in vertebrates or assist cell movement in unicellular organisms [32,33,34,35,36,37,38,39].

## 3. Cilia Disassembly and Its Regulation

Depending upon the cell type, culture conditions, or both, there are two different ways by which a cell can undergo ciliary loss: (1) cilia disassembly, where the length of the primary cilia is gradually reduced [40,41,42,43], and (2) shedding, where the primary cilia are instantly cut off from the main cell body [44,45,46,47,48]. However, in some lower organisms, the ciliary loss can also be mediated by the internalization of the whole axoneme. In such cases, within seconds, the whole cilium is retracted inside the cell and later gets disintegrated under an hour [49,50]. It is still unclear how a cell chooses to undergo any of the processes mentioned above for its ciliary loss and whether and how the route of ciliary loss impacts cellular functions. While the importance of cilia shedding has been understudied in human diseases, dysregulation of the cilia disassembly has been implicated in multiple diseases. Therefore, in recent years, significant efforts have been made to understand how ciliary disassembly is regulated.

### 3.1. Model Systems Employed to Study Cilia Disassembly Process

Pioneering studies in the green algae *Chlamydomonas reinhardtii* revealed some of the important molecular players during ciliary loss. Since then, it has been a well-established model organism to study ciliary disassembly. *Chlamydomonas* is a single-cell green alga with two long flagella structurally similar to vertebrate motile cilia and is known to undergo de-flagellation. Based on the stimulus, *Chlamydomonas* can undergo gradual flagellar disassembly or shedding. It is shown that exposure to low pH (4.5) induces a rapid de-flagellation at the distal ends of the basal bodies [51], analogous to shedding in mammalian cells. In contrast, after gamete fertilization, quadri-flagellated *Chlamydomonas* zygote tends to resorb its flagellates gradually in a process analogous to the cilia disassembly process in mammalian cells [52]. Altering the culture conditions such as tonicity or calcium concentration can cause flagellar disassembly [44,53]. Collectively, these studies highlight the use of *Chlamydomonas* as a model system to study ciliary loss. Because of the easiness to observe flagella under the brightfield microscope, suitability for genetic manipulations, and ability to isolate flagella for protein purification, studies using *Chlamydomonas* have led to the discovery of critical proteins required for the cilia disassembly process. These proteins include Aurora-A (AurA), NIMA (never in mitosis A)-related protein kinase (NEK), and many more [52,54,55,56,57].

In recent years, researchers have adopted the use of mammalian cell lines to delineate the process of cilia disassembly. As mentioned earlier, the presence of cilia is inversely correlated with cell cycle progression. It is shown that cells tend to assemble their cilia during cellular quiescence (G0/G1 phase) and disassemble their cilia when stimulated to enter the cell cycle (S/G2/M phase) [58]. Hence, to study cilia disassembly in cell culture models, cells are first synchronized in the G0/G1 phase of the cell cycle via serum starvation and then are induced to re-enter the cell cycle via serum re-addition. Entering the S phase triggers ciliary disassembly or shedding [50,52]. Although all ciliated cells are considered to undergo ciliary loss, not all cells are ideal for studying the process of cilia disassembly using serum depletion/re-addition protocols in vitro. In general, a suitable cell line for studying cilia disassembly should satisfy the following criteria. First, cells should achieve a high level of synchronization at G0/G1 phase (>75%) upon serum starvation. Second, a significant percentage of cells (>50%) should have cilia at ~80% confluence following serum starvation. This is important as cells that require 100% confluence for efficient ciliation cannot undergo cilia disassembly upon serum re-addition, as cells are often contact-inhibited and cannot progress to the S phase. Third, cells should express all essential proteins for cilia assembly and disassembly. Traditionally, cell lines such as mouse fibroblast NIH3T3 cells or human retinal pigmented epithelial (RPE1) cells have been extensively used for studying cilia dynamics, especially cilia disassembly, as they fulfill all the criteria mentioned above. In vivo, the assessment of cilia disassembly is extremely challenging as it is difficult to synchronize cell cycle re-entry in animal models. Combinations of appropriate cell cycle (i.e., FUCCI system) and ciliary markers (Arl13b reporter) have been employed to study ciliary disassembly in real-time in zebrafish [59] and mice [60]. However, as discussed previously [60], several limitations are associated with these approaches. Overall, much of our current knowledge on mechanisms of ciliary loss has been obtained essentially from experiments conducted in cell culture systems using established cell lines and unicellular organisms such as *Chlamydomonas*.

### 3.2. Cellular Processes That Govern Transient or Permanent Modes of Ciliary Disassembly

Ciliary loss can be transient or permanent. It can occur in a transient manner when cilia assemble and disassemble depending upon their cell cycle stage or upon induction of cellular differentiation or stress. Permanent ciliary loss occurs when ciliated stem cells differentiate into non-ciliated cells. In either case, a wide variety of internal and external cues co-ordinate to carry out the ciliary disassembly. Downstream signaling cascades activated upon such cues can be distinct and cell-type specific.

Cell cycle: As discussed earlier, cells assemble their cilia when they exit from the cell cycle and disassemble it upon cell cycle entry [58]. In most cell types, primary cilia attain their full length in G0/G1 phase, and a shorter version can be detectable in S and G2 phases, depending on the cell type. Complete resorption is observed prior to mitotic entry. Tucker et al. [41,42] were the first to report that the cell cycle induces cilia disassembly using 3T3 fibroblast cells. In their experiments, they showed that cells under serum starvation display fully formed cilia, and upon serum stimulation, undergoes cilia disassembly in two phases. The first phase of cilia disassembly occurs immediately after serum re-addition (~within 1–2 h), followed by a plateau in ciliation (up to 10 h). Interestingly, they observed a marginal increase in ciliation (~10–20 h) prior to the final or second phase of disassembly (~20–30 h), after which cilia became almost undetectable. Several research groups, including ours, have also reported a similar pattern of cilia disassembly using other cell lines such as RPE1 cells, inner medullary collecting duct cells (IMCD) cells, or mouse embryonic fibroblast (MEFs) [43,61,62]. Similar to 3T3 fibroblast, it was observed that these cells undergo cilia disassembly when stimulated to re-enter the cell cycle. However, the length of cilia and the disassembly rate varied based on the cell type and growth conditions.

Cell Differentiation: Primary cilia are shown to be important for the maintenance and/or differentiation of stem cells such as mesenchymal stem cells, neural stem cells, etc. [63]. Interestingly, studies have shown that ciliary loss can occur when cells undergo differentiation both in mammalian cells and unicellular organisms. It is shown that when epithelial cells or mesenchymal fibroblasts differentiate to myofibroblast, cilia are completely disassembled and thereby significantly alter Hedgehog and platelet-derived growth factor (PDGF) signaling pathways that are known to regulate the cell differentiation process [64,65]. Ciliary loss has also been reported during different stages of protozoa, algae, and fungi [50]. In mammals, it is shown that delayed cilia disassembly can alter the fate of neural progenitor cells from self-renewal to premature differentiation and can cause microcephaly [66]. Given that cilia length has been positively correlated with activation of key signaling pathways (such as Sonic Hedgehog or PDGF) that are involved in stem cell differentiation, it will be interesting to evaluate the effect of changes in cilia disassembly rate on the lineage commitment or differentiation of various stem cells.

Cellular stress: Multiple cellular stressors, including heat shock, chemical exposure, and mechanical stress, have been reported to induce ciliary loss [67,68,69]. Studies in *Chlamydomonas* have shown that flagellar length increases upon reducing osmolarity, whereas flagellar length decreases upon increasing the osmolarity in the culture medium [44]. In addition, chemical treatment of sodium pyrophosphate or mechanical shearing stress also induces complete flagellar resorption in *Chlamydomonas* [54]. In mammalian NIH3T3 cells, a 30-min heat shock treatment results in the complete removal of cilia in approximately half of the ciliated cells [69]. Further, human umbilical vein endothelial cells (HUVECs) results in ciliary loss under laminar shear stress [67]. Similarly, fluid flow-based shear stress causes ciliary loss in the cells present in the eye’s trabecular meshwork [70]. Together, these data suggest that cells are equipped to respond to different environmental stress signals by inducing ciliary loss. However, whether the stress-induced ciliary loss is functionally coupled to other cellular processes such as cell migration, proliferation or differentiation is yet to be determined.

### 3.3. Molecular Events Regulating Ciliary Disassembly

**Figure 2:**
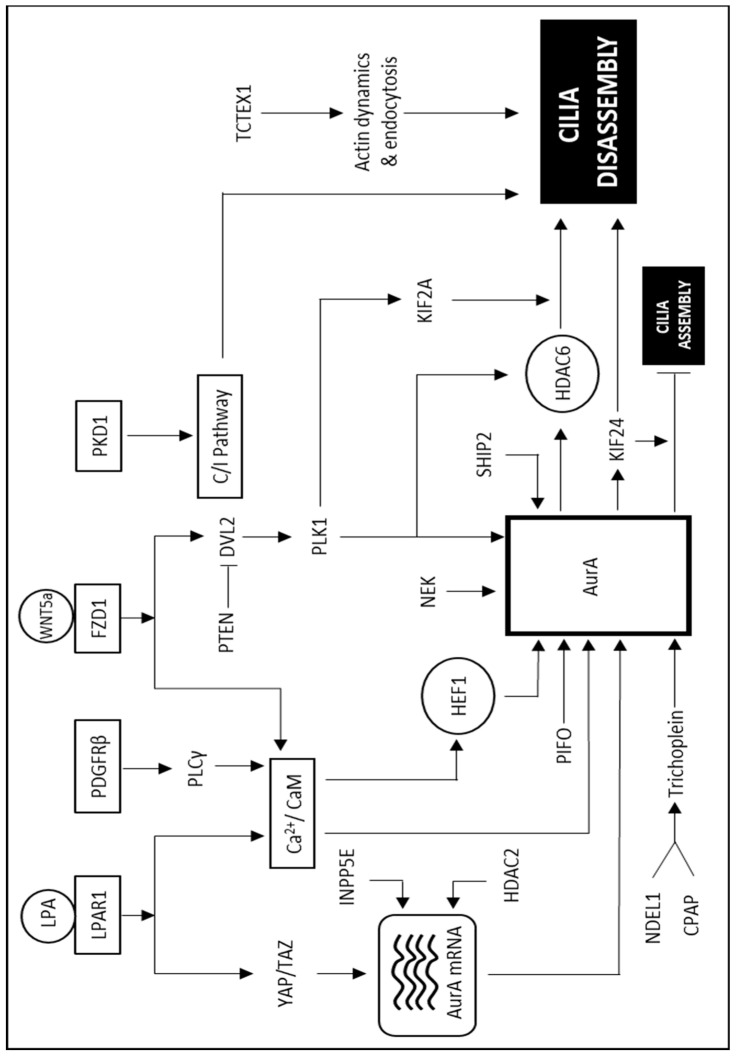
Mechanisms of cilia disassembly. Major signaling cascades and molecular players regulating the process of cilia disassembly.

Several signaling pathways and molecular players are implicated in regulating the process of cilia disassembly (Figure 2). Based on the current literature, the molecular events regulating the process of cilia disassembly can be broadly categorized into (1) activation of AurA kinase and deacetylation of microtubules, (2) depolymerization of microtubules, and (3) ciliary membrane remodeling and inhibition of cilia assembly.

#### 3.3.1. Regulation of AurA Kinase and Deacetylation of Microtubules

As discussed previously, mechanistic understanding of cilia disassembly was first studied in lower eukaryotes, such as Chlamydomonas, which can undergo either gradual flagellar resorption or shedding depending upon the stimulus. Several studies in Chlamydomonas showed that Chlamydomonas aurora-like protein kinase (CALK)-mediated axoneme destabilization is crucial for flagellar resorption, and its depletion results in impaired flagellar resorption. Studies have shown that CALK is phosphorylated immediately after stimulation of flagellar resorption [56]. Several factors have been reported to induce CALK phosphorylation including, (1) sodium pyrophosphate treatment, which triggers flagellar shortening [54], and (2) post gamete fertilization, where quadri-flagellated zygote undergoes gradual shortening [52]. Subsequent studies have shown that CALK phosphorylation at T193 is mainly responsible for the process of flagellar resorption [71]. CALK is indistinctly related to the human AurA kinase, which is a centrosomal kinase that regulates mitotic entry in mammalian cells [72,73]. Once the role of CALK in flagellar resorption was established, the involvement of AurA kinase in the cilia disassembly process was assessed. AurA kinase was shown to be a central player in regulating cilia disassembly in mammalian cells via human enhancer of filamentation 1 (HEF1)/AurA/histone deacetylase 6 (HDAC6) axis [40]. This role of AurA was entirely independent of its role in mitosis, as these mechanistic studies were performed in cells emerging out of the G0/G1 phase. Upon serum stimulation, HEF1, a scaffolding protein, has been shown to bind and stabilize [74] AurA kinase at the base of the cilia. This HEF1 and AurA kinase association has been shown to play a major role in AurA-mediated phosphorylation of HDAC6. HDAC6 further deacetylates α-tubulin and cortactin, which in turn facilitates ciliary disassembly by different mechanisms [75]. For instance, HDAC6-mediated deacetylation of α-tubulin [76,77] leads to instability of axoneme microtubules, whereas HDAC6-mediated deacetylation of cortactin increases its binding to F-actin, resulting in enhanced actin polymerization, which then collectively aid in the process of cilia disassembly. More recently, histone deacetylase 2 (HDAC2) has been shown to promote the cilia disassembly process [78]. After the discovery of the importance of the HEF1/AurA/HDAC6 axis in cilia disassembly regulation, multiple studies have been performed to characterize the regulation of the HEF1/AurA/HDAC6 axis and identify its upstream and downstream regulators that are key players in orchestrating the process of cilia disassembly.

Among the different regulatory players, calcium (Ca^2+^) and calmodulin (CaM) are important for forming the HEF1-AurA complex and activating AurA kinase at the basal body during the ciliary disassembly process [40,79]. PDGFRβ has also been shown to promote cilium disassembly by activating phosphoinositide phospholipase C gamma, which causes the release of intracellular Ca^2+^ and activation of CaM and AurA [80]. Collectively, these data show that signaling pathways that cause the release of intracellular Ca^2+^ and activation of CaM could indirectly lead to AurA activation and thereby promote ciliary disassembly.

Non-canonical WNT signaling has also been shown to regulate AurA. Specifically, WNT5a activates casein kinase 1 epsilon (CK1ε), which then phosphorylates conserved S143 and T224 sites of dishevelled2 (DVL2) and facilitates a physical interaction between DVL2 and polo-like kinase 1 (PLK1) [81]. The DVL2–PLK1 complex enhances the ability of DVL2 to interact with SMAD3 and thereby competitively inhibits SMAD3-HEF1 interaction, causing an increase in HEF1 levels [81]. This increase in HEF1 levels aids in promoting HEF1-AurA-HDAC6 axis and cilia disassembly. Further studies have shown that the DVL2 S143A mutant disrupts the interaction of DVL2-PLK1 and reverses the increased cilia disassembly seen upon WNT5a stimulation [81]. Interestingly, tumor suppressor PTEN suppresses CK1ε-dependent phosphorylation of DVL2, which otherwise promotes cilia disassembly via PLK1/AurA signaling [82]. In a separate study, PLK1 has also been shown to promote cilia disassembly via HDAC6 activation. It is shown that pericentriolar material 1 (PCM1), a centriolar satellite protein, undergoes cyclin-D kinase 1-dependent phosphorylation and recruits PLK1 to the pericentriolar matrix. Primary cilia resorption is dependent on the kinase activity of the pericentriolar matrix localized PLK1, where PLK1 induces HDAC6 activation [83]. This, in turn, leads to ciliary microtubule deacetylation and disassembly. PLK1 has also been implicated in protein trafficking by phosphorylating the transition zone protein nephrocystin-1 [84]. Centrosomal protein of 55 kD (CEP55) is shown to facilitate the recruitment of chaperonin containing TCP1 chaperonin complex to AurA, stabilizing AurA and promoting ciliary disassembly [85]. Studies from our lab have shown that the centrosomal integrity/mitotic surveillance (CI) pathway comprising USP28, p53, and 53BP1 plays a significant role in cilia disassembly downstream of polycystin genes (*Pkd1* or *Pkd2*) [43]. It was shown that the loss of polycystins activates the CI pathway causing a delay in the rate of ciliary disassembly. The mechanisms by which activation of p53 slows down ciliary disassembly are consistent with its role as a tumor suppressor. The exact mechanism by which p53 functionally interacts with the disassembly machinery warrants further investigation. Since the polycystin complex (PKD1 and PKD2) mediates WNT/Ca^2+^ signaling [86], it will be interesting to know further if WNT/Ca^2+^ signaling and CI pathway crosstalk during the process of cilia disassembly. Furthermore, whether WNT5a stimulation or PTEN can affect the association of PLK1 with PCM1 or nephrocystin-1 remains a subject of further investigation.

Transcriptional regulation of AurA is also shown to be important in regulating the process of ciliary disassembly, apart from its direct activation or stabilization, as discussed above. Studies show that inositol polyphosphate-5-phosphatase E (INPP5E) regulates AurA protein levels by increasing AurA transcript levels by AKT activation, which can impact the ciliary disassembly process [87]. Fibroblast growth factor receptor 1 oncogene partner is also shown to affect cilia disassembly by regulating AurA expression [88]. AurA transcription is also shown to be regulated by different signaling pathways, including, ERK-responsive Ets pathway, STAT5, estrogen/GATA3, HIF1, etc. [89]. As the majority of these studies were performed in cancer cells, the transcriptional regulation of AurA was mainly associated with its role in mitosis and cell proliferation. However, whether these signaling pathways affect cilia disassembly by regulating AurA and its implications on cancer cells in the G0/G1/S phase of the cell cycle is largely unexplored.

Besides direct activation, stabilization, or transcriptional regulation of ciliary disassembly factors, cellular localization can also impact the cilia disassembly process. It is shown that SH2-containing phosphatidylinositol 3,4,5-trisphosphate 5-phosphatase implicated in phosphoinositide signaling can relocate AurA and HEF1 from the apical to the basolateral surface of epithelial cells and thereby promote cilia formation in epithelial cells toward tubular lumen [90]. Proteins that aid in the stabilization or activation of AurA also gets localized near the basal body. Pitchfork (PIFO) is known to be localized at the basal body and can activate AurA during the process of ciliary disassembly [91]. Interestingly, it is suggested that AurA belongs to a primary cilium disassembly complex (CDC) containing CPAP, NDE1, and OFD1 [66]. This complex, upon mitogenic signaling, assembles near the basal body and aid in the process of cilia disassembly. Further studies are required to evaluate if cilia disassembly associated proteins such as PIFO, PCM1, or PLK1 are a part of or transiently interact with the CDC complex during the ciliary disassembly process.

Recently, Hu H and colleagues [92] have identified lysophosphatidic acid (LPA) as an extracellular cue that initiates the process of cilia disassembly. For years, serum re-stimulation of serum-starved cells has been used to induce cilia disassembly. However, the extract molecular signal present in serum triggering cilia disassembly was not known. Hu H and colleagues showed that LPA present in serum is primarily responsible for inducing cilia disassembly. LPA binds to its receptor LPA receptor 1 (LPAR1) and promotes the transcription and phosphorylation of AurA through activating YAP/TAZ and Ca^2+^/CaM pathways, respectively. Depletion of LPA or LPAR1 results in slower cilia disassembly and longer cilia. It would be further interesting to test whether LPA stimulation crosstalks with non-canonical WNT signaling or is responsible for regulating any of the cilia disassembly associated proteins discussed above. Collectively, all these studies highlight the central role of AurA in ciliary disassembly.

#### 3.3.2. Depolymerization of Microtubules

Microtubule (MT) depolymerization follows AurA-induced MT destabilization. Seminal studies in Chlamydomonas have identified NEKs as the kinases controlling both cell cycle progression and flagellar disassembly or shedding [55,57]. Consecutively, studies in mammalian cells also highlighted the importance of NEK kinases in cilia disassembly. Specifically, it is shown that the cells depleted with NEK2 are not able to disassemble their cilia before the onset of mitosis. In parallel, cells that have overexpression of catalytically active NEK2A display shorter cilia. Since cells depleted with NEK2 did not cause any changes in T288 phosphorylated AurA levels, it was suggested that NEK2 works downstream of AurA in the cilia disassembly process, which was shown in subsequent studies [93,94]. NEK1, another NEK kinase, is also shown to play a role in cilia disassembly. In addition, an E3 ligase, anaphase-promoting complex (APC), and its co-activator CDC20, both localized at the basal body, can target NEK1 for its proteasomal degradation and thereby regulate the process of ciliary disassembly [95].

Kinesin family member 24 (KIF24) and kinesin family member 2A (KIF2A) belonging to the kinesin-13 family of motor proteins are shown to be important in the process of MT depolymerization necessary for ciliary disassembly [94,96,97]. Although most kinesins are involved in intracellular transport, some kinesins possess MT-depolymerizing activities to control MT dynamics [98,99]. Since kinesins interact with MTs, they were suspected of playing a role in cilia formation as well as resorption [100]. It was shown that downstream of AurA, NEK2 can activate KIF24 and thereby trigger the MT depolymerization necessary for ciliary disassembly [94]. Since NEK2 is expressed in the S and G2 phases of the cell cycle, it ensures that the KIF24 dependent MT depolymerization and subsequent ciliary disassembly is conducted before cells enter into mitosis [94]. KIF24 has also been shown to recruit the CP110/CEP97 centriole capping complex, which prevents axoneme growth and generally indicates the completion of cilia disassembly [96]. Hence, KIF24 plays a dual role of actively depolymerizing the axoneme as well as inhibiting the cilia growth. These dual roles of promoting cilia disassembly and inhibiting cilia assembly suggest that NEK2 and KIF24 ensure that the ciliary loss is irrevocable once the cells enter the cell cycle. In conjunction with KIF24, KIF2A can also depolymerize MTs [97]. KIF2A also participates in cilia disassembly during the G2 phase of the cell cycle, where it is found to be localized at the centrioles. PLK1, which is considered a G2/M phase kinase, is shown to phosphorylate KIF2A, causing MT depolymerization and promoting ciliary disassembly [97]. Since PLK1 is known to phosphorylate other kinesin-13 family members such as KIF2B or KIF2C in order to mediate proper chromosome segregation and spindle assembly [101,102], it will be interesting to see if a similar role of PLK1-KIF2B/2C exists in the context of cilia disassembly process during G0/G1 phase of the cell cycle.

#### 3.3.3. Ciliary Membrane Remodeling and Inhibition of Cilia Assembly

In parallel to axoneme destabilization and depolymerization, remodeling of the ciliary membrane and cytoskeleton, both at the base and tip of the shortening primary cilium, is also necessary during disassembly. Recent evidence suggests one of the earliest steps in cilia disassembly involves a “chopping” mechanism at the tip of the disassembling cilia. As a result, ciliary vesicles (CVs) are excised and released from the tip of the cilium soon after stimulation of the cilia disassembly process. The underlying mechanism involves AurA activation, which decreases INPP5E levels and, thereby, results in intraciliary redistribution of phosphatidylinositol 4,5-biphosphate (PI(4,5)P2). PI(4,5)P2, together with actin regulators, marks the specific location of CV excision by local induction of intra-ciliary actin polymerization [103]. Interestingly, proteomic analyses of CVs showed an abundance of intraflagellar transport complex B (IFT-B) rather than intraflagellar transport complex A (IFT-A), suggesting that CVs preferentially remove IFT-B from primary cilia, limiting cilia growth and indirectly promoting cilia disassembly [103].

The base of the cilium is surrounded by the ciliary pocket, a structure rich in actin network density. Remodeling of the ciliary pocket membrane during disassembly is mediated by TCTEX-1 (DYNLT1, dynein light chain-1), which is activated by phosphorylation at T94. TCTEX-1 was initially described as a light chain subunit of cytoplasmic dynein [104], and the role of phosphorylated T94 TCTEX-1 in regulating cilia disassembly has been described in cortical neuronal progenitors cells [105]. Phosphorylation of TCTEX-1 at T94 is required for both cilium resorption and entry into the S phase of the cell cycle. Mechanistically, phosphorylation of TCTEX-1 at T94 leads to the dissociation of TCTEX-1 from the dynein complex, facilitating cilia resorption. TCTEX-1 activates F-actin polymerization triggering a cascade of events that coordinately lead to cilia resorption and cytoskeletal rearrangement [105]. The active form of TCTEX-1 binds to annexin A2, actin-related protein 2/3 complex subunit 2, and cell division control protein 42, which actively regulates actin dynamics and clathrin-dependent endocytosis at the ciliary base, thus mediating the remodeling of the ciliary pocket membrane during cilia disassembly [106]. Inhibition of phosphorylation of TCTEX-1 at T94 induces neuronal differentiation instead of proliferation, highlighting its importance in coupling ciliary disassembly and cell cycle progression. More recently, it has also been shown that exocysts localized near the ciliary base assist in recycling the resorbed cilia and en route the ciliary components back to the cell surface. Whether and how this process affects ciliary disassembly is a subject of further investigation [107].

In addition to actively promoting the activity of cilia disassembly pathways, cilia disassembly can be indirectly promoted by suppressing the activity of ciliary assembly pathways. The NDEL1-Trichoplein-AurA axis [108,109], NEK2-KIF24, and the CPAP-Trichoplein-AurA axis are known to inhibit cilia assembly when the cell enters the cell cycle (S/G2/M phases) [66,109]. Interestingly, LPA-LPAR-1, shown to induce cilia disassembly, can also inhibit ciliogenesis. It is shown that LPA activates PI3K/Akt kinase pathway via the LPAR1 receptor, which further blocks RAB11a-RABIN8 binding and inhibits preciliary trafficking and ciliogenesis initiation [110]. These pathways collectively dampen the machinery required for cilia assembly and ensure that no ectopic ciliary assembly occurs during ciliary loss and entry into the cell cycle.

## 4. Concluding Remarks and Futures Directions

Our understanding of the mechanisms of ciliary disassembly has been gradually increasing. For years, serum-induced ciliary loss has been extensively used as a workhorse to obtain mechanistic information on ciliary disassembly and/or shedding. However, the presence of various growth factors in the serum, overlap in the timing of ciliary disassembly and cell cycle re-entry, and non-physiological manner to induce cell cycle arrest have posed difficulties in teasing out the ciliary disassembly-specific factors from the signals that are associated with the cell cycle. The identification of LPA [92] as the primary stimulus for cilia disassembly initiation has refined existing experimental models to evaluate the process and sequence of events in cilia disassembly. Thus far, several studies have helped us accomplish a basic understanding of the molecular players regulating the process of ciliary loss involving AurA activation, axonemal microtubules depolymerization, and ciliary membrane remodeling. However, many important questions are yet to be addressed. Our knowledge of the mechanisms and regulation of ciliary shedding as a distinct mode of ciliary loss is fragmented. The functional consequence of undergoing cilia disassembly versus shedding is yet to be determined. Understanding the biological roles(s) of cilia disassembly also remains an open question. In this regard, while it will be challenging to synchronize cells in vivo to study the biological roles of accelerated or suppressed disassembly rates, naturally occurring or experimentally induced mutations in genes involved in the disassembly process could be informative. For instance, recent studies have shown that loss of polycystin gene(s), known to cause autosomal dominant polycystic kidney disease (ADPKD), results in delayed cilia disassembly and displays abnormally elongated cilia in mouse models as well as in ADPKD patients [43,111]. Combined with the increased cell proliferation seen in almost all cases of ADPKD in mice and patients, it appears that uncoupling of ciliary disassembly from the cell cycle may be prevalent and perhaps, one of the underlying causes of ADPKD. Given that ablation of cilia corrects the ADPKD phenotype in mouse models [112], elongated cilia resulting from cilia disassembly defects could be considered as a predisposing factor in ADPKD. Consistently, pharmacologic acceleration of ciliary disassembly suppresses cystic growth, whereas deletion of HEF1 in PKD1-null kidneys exaggerates cystic growth [113,114]. Collectively, all these studies suggest that one of the biological roles of ciliary disassembly in the kidney may be the maintenance of normal tubular architecture and prevention of cyst formation/progression. Furthermore, cilia disassembly factors such as INPPE5 are known to cause Joubert and mental retardation, truncal obesity, retinal dystrophy, and micropenis (MORM) syndromes, which are considered ciliopathies [115]. Recently discovered LPA/LPAR1 signaling that initiates cilia disassembly in serum-starved cells is also shown to be important in neurogenesis. It is shown that the deletion of LPAR1 results in elongated cilia and decreased proliferation in neural progenitor cells, resulting in defective neurogenesis [92]. Likewise, many cilia disassembly factors have been implicated in multiple ciliopathies and cancers and have been reviewed previously [4,116,117,118,119,120,121]. However, whether their role in such diseased states is entirely or partly via cilia disassembly defects is a subject of further investigation. Regardless, the studies mentioned above collectively highlight the biological relevance of the cilia disassembly process. In parallel, many studies have also implicated that cilia disassembly is well synchronized with the cell cycle, and it acts as a checkpoint for S phase entry [58,60,122]. However, how the cilia disassembly and cell cycle progression are coupled together still remains poorly understood. Whether defective cilia disassembly results in dysregulation of cellular processes such as cell cycle or cell differentiation is still unclear and warrants further investigation; it could potentially explain several developmental defects seen in a broad spectrum of ciliopathies [4,23].

From a technical standpoint, since cellular processes such as cell cycle and differentiation are tightly coupled with cilia dynamics, many proteins that regulate these processes are found to affect ciliary length. In addition, certain proteins might play a role in both cilia assembly and disassembly, possibly via different mechanisms. Hence, in order to tease out the role of such proteins in cilia dynamics, future studies may require the use of more refined techniques such as auxin-inducible degron-mediated protein degradation and use of proteolysis-targeting chimera to temporarily and spatially knockdown protein levels, especially during cilia disassembly. Additionally, tracking the cilia disassembly of single cells with confocal and time-lapse microscopy will also help decipher the mechanisms and sequence of events during cilia disassembly.

Defects in cilia resulting in either shorter or longer cilia are associated with a wide variety of ciliopathies, highlighting the importance of studying the mechanism(s) underlying the cilia dynamics. Understanding the regulation and molecular players involved in ciliary disassembly will bring us one step closer to understanding various ciliopathies and may reveal targets for drug therapy, which is currently lacking.

## Data Availability

Not applicable.
